# Compositional Features of HDL Particles Interact with Albuminuria to Modulate Cardiovascular Disease Risk

**DOI:** 10.3390/ijms20040977

**Published:** 2019-02-23

**Authors:** James P. Corsetti, Stephan J. L. Bakker, Ronald T. Gansevoort, Eke G. Gruppen, Margery A. Connelly, Charles E. Sparks, Robin P. F. Dullaart

**Affiliations:** 1 Department of Pathology and Laboratory Medicine, University of Rochester School of Medicine and Dentistry, Rochester, NY 14642, USA; charles_sparks@urmc.rochester.edu; 2 Department of Nephrology, University of Groningen and University Medical Center Groningen, 9700 RB Groningen, The Netherlands; s.j.l.bakker@umcg.nl (S.J.L.B.); r.t.gansevoort@umcg.nl (R.T.G.); e.g.gruppen@umcg.nl (E.G.G.); 3 Department of Endocrinology, University of Groningen and University Medical Center Groningen, 9700 RB Groningen, The Netherlands; r.p.f.dullaart@umcg.nl; 4 Laboratory Corporation of America Holdings (LabCorp), Morrisville, NC 27560, USA; connem5@labcorp.com

**Keywords:** albuminuria, urinary albumin excretion, HDL, apolipoprotein A-I, HDL subfractions

## Abstract

Lipoproteins containing apolipoprotein B modify associations of elevated urinary albumin excretion (UAE) with cardiovascular disease (CVD). Additionally, it is known that elevated UAE alters high-density lipoprotein functionality. Accordingly, we examined whether HDL features might also modify UAE-associated CVD. Multivariable Cox proportional-hazards modeling was performed on participants of the PREVEND (Prevention of Renal and Vascular Endstage Disease) study at the baseline screening with standard lipid/lipoprotein analyses and, three-to-four years later (second screen), with nuclear magnetic resonance lipoprotein analyses focusing on HDL parameters including HDL particle (HDL-P) and apolipoprotein A-I concentrations. These were used with UAE and derived measures of HDL apoA-I content (apoA-I/HDL-C and apoA-I/HDL-P) in risk models adjusted for gender, age, apoB, diabetes, past CVD history, CRP and GFR. Interaction analysis was also performed. Baseline screening revealed significant associations inverse for HDL-C and apoA-I and direct for apoA-I/HDL-C. The second screening demonstrated associations inverse for HDL-P, large HDL-P, medium HDL-P, HDL size, and apoA-I/HDL-P. Significant interactions with UAE included apoA-I/HDL-C at the baseline screening, and apoA-I/HDL-P and medium HDL-P but not apoA-I/HDL-C at the second screening. We conclude that features of HDL particles including apoA-I/HDL-P, indicative of HDL apoA-I content, and medium HDL-P modify associations of elevated UAE with CVD risk.

## 1. Introduction

Elevated urinary albumin excretion (UAE) is well known to be associated with cardiovascular disease (CVD) risk [1,2,3,4,5,6,7,8]. Furthermore, it is likely that coexisting atherogenic lipoprotein abnormalities in individuals with elevated UAE modify CVD risk in this setting [9,10], a notion that is consistent with reports of elevated levels of plasma atherogenic apolipoprotein B (apoB) particles in individuals with proteinuria [11,12,13,14,15]. In this case, elevated UAE and atherogenic apoB particles may act independently or together in modulating CVD risk. If acting in concert, this might be indicative of shared pathogenic mechanisms leading to CVD risk. Thus, in an earlier report from the Prevention of Renal and Vascular Endstage Disease (PREVEND) study [10], statistical interactions of elevated UAE with multiple apoB-containing lipoprotein fractions were demonstrated, leading to the conclusion that elevated UAE and atherogenic apoB lipoproteins characteristic of albuminuric subjects likely do share common pathogenic mechanisms leading to CVD.

In contrast, for high-density lipoprotein (HDL) in individuals with nephrotic syndrome and chronic kidney disease (CKD), HDL cholesterol (HDL-C) is within or below normal limits [16]. Additionally, in these individuals, profound changes in the structure and protective features of HDL particles may occur, including impairments in reverse cholesterol transport, as well as anti-inflammatory, antioxidant, and antithrombotic functionalities [16]. Accordingly, in the current study, we hypothesized that various attributes of HDL particles might demonstrate modulation of the CVD risk associated with elevated UAE, which is potentially indicative of a commonality of pathogenic pathways. To address this question, we investigated 6,286 participants of the PREVEND study using nuclear magnetic resonance (NMR) lipoprotein analyses with a particular focus on HDL-associated parameters along with multivariable Cox proportional hazards risk modeling as a function of UAE and HDL-associated parameters by NMR to determine risk associations and potential interactions between UAE and HDL-associated parameters that might underlie CVD risk.

## 2. Results

### 2.1. Study Group Characterization

**Figure 1:**
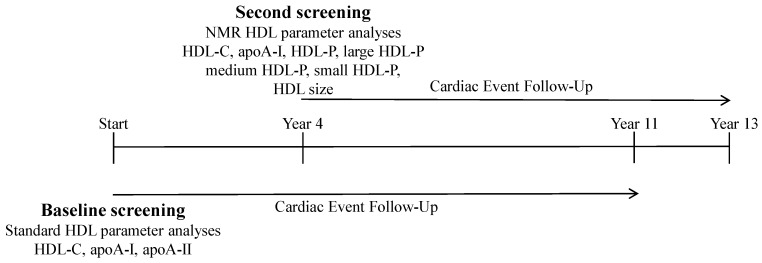
Overview of study design.

Figure 1 gives an overview of the temporal (baseline screening and second screening) and parameter analysis scheme for the study. Table 1 gives a summary for subjects at the baseline evaluation of clinical and laboratory parameters stratified according to UAE as follows: normoalbuminuria (<30 mg/24 h), microalbuminuria (30 to 300 mg/24 h), and macroalbuminuria (>300 mg/24 h). Unadjusted ANOVA and chi-square testing results demonstrated significant differences among the UAE groups for all parameters of Table 1 except for ethanol use. Results of post-hoc statistical comparisons revealed the following: for the normoalbuminuric versus microalbuminuric groups, differences in all parameters except for apoA-II; for the normoalbuminuric versus macroalbuminuric groups, differences in all parameters except for apoA-I and apoA-II/HDL-C; and for the microalbuminuric versus macroalbuminuric groups, differences in BMI, systolic blood pressure, creatinine, eGFR, apoA-II, triglycerides, and glucose. With adjustment of ANOVA models for gender and age and further adjustment for gender, age, apoB, diabetes, past CVD history, and eGFR; all lipid and lipoprotein markers remained significantly associated with the UAE groups except for apoA-I, cholesterol, and LDL-C.

### 2.2. CVD Risk Associations at Baseline Screening of UAE and HDL-Associated Parameters

To examine potential associations of UAE and HDL-associated parameters with CVD risk, Cox multivariable proportional hazards models adjusted for gender and age were formulated with time to event (from the time of baseline evaluation and extending for median follow-up time of 10.5 years) as a function of UAE along with separate entry of HDL-C, apoA-I, apoA-II, apoA-I/HDL-C (crude estimate of apoA-I HDL particle content), and apoA-II/HDL-C (crude estimate of apoA-II HDL particle content). The results (Table 2) demonstrated in each case significant direct risk association of UAE along with independent inverse risk associations with HDL-C, apoA-I, and apoA-II; and independent direct risk associations with apoA-I/HDL-C and apoA-II/HDL-C.

Interactions between UAE and the HDL-associated parameters were also examined by inclusion of corresponding interactions terms into the gender- and age-adjusted models. Results (Table 2) demonstrated significant interaction with UAE only for apoA-I/HDL-C. Moreover, when models were concurrently adjusted for gender, age, apoB concentration, diabetes, past CVD history, CRP concentration, and eGFR; again significant interaction with UAE was demonstrated only for apoA-I/HDL-C (Table 3).

### 2.3. Lipoprotein NMR Analyses

Approximately three to four years after study initiation, blood and urine were again collected on a new screening round in which UAE determinations and NMR lipid and lipoprotein analyses were performed on the subjects of Table 1. The results from the NMR analyses, according to albuminuria status, are given in Table 4 for concentrations of total HDL particles (HDL-P), large HDL particle (large HDL-P), medium HDL particles (medium HDL-P), small HDL particles (small HDL-P), mean HDL size, and concentrations of HDL-C and apoA-I, as well as calculated values of apoA-I/HDL-C and apoA-I/HDL-P. From Table 4, trends in parameters with increasing UAE status are demonstrated. Unadjusted ANOVA results demonstrated significant differences among the UAE groups for all parameters of Table 4. Table 4 also presents results of post-hoc statistical comparisons between albuminuria groups. With adjustment of ANOVA models for gender and age and further adjustment for gender, age, apoB, diabetes, past CVD history, and eGFR, all NMR lipoprotein markers remained significantly associated with the UAE groups except for medium and small HDL-P and the ratio of apoA-I/HDL-P.

Lastly, multiple regression analysis with total apoA-I concentration as a function of large, medium, and small particles was used to estimate the mean number of apoA-I molecules/particle for the three subfractions (Appendix A). Results were as follows: large HDL: 4.49 ± 0.014, *p* < 10^−6^; medium HDL: 2.55 ± 0.007, *p* < 10^−6^; and small HDL: 1.68 ± 0.003, *p* < 10^−6^).

### 2.4. CVD Risk Associations at Second Screening of UAE and HDL-associated Parameters

Results from Table 2 revealed significant interaction for UAE with the apoA-I/HDL-C ratio, a crude measure of the apoA-I content of HDL particles. However, since NMR lipoprotein analysis provides total HDL particle concentrations and apoA-I concentrations, it thus makes it possible to calculate the mean number of apoA-I molecules per HDL particle. To examine potential associations of UAE and HDL-associated NMR parameters with CVD risk, especially as related to the apoA-I content of HDL particles, Cox multivariable proportional hazards models (from the time of the second screening round and extending for median follow-up time of 8.3 years) adjusted for gender and age were formulated with time to event as a function of UAE along with a separate entry of each of the nine parameters of Table 4. The results (Table 5) demonstrated, in each case, significant direct risk association of UAE along with independent inverse risk associations with HDL-P, large HDL-P, medium HDL-P, HDL size, HDL-C, apoA-I, and the apoA-I/HDL-P; and independent direct risk association for the apoA-I/HDL-C ratio.

Interactions between UAE and the HDL-associated NMR parameters were also examined by including corresponding interaction terms into the gender and age adjusted models. The results (Table 5) demonstrated significant interaction for UAE with medium HDL-P (*p* = 0.029) and with the apoA-I/HDL-P ratio (*p* = 0.039) and close approach to significance for HDL-C (*p* = 0.060). Results were essentially the same when models were concurrently adjusted for gender, age, apoB concentration, diabetes, past CVD history, CRP, and eGFR (Table 6). The results suggest a role for the apoA-I content of HDL particles and medium HDL-P and potentially for HDL-C in the association of UAE with CVD risk.

**Figure 2:**
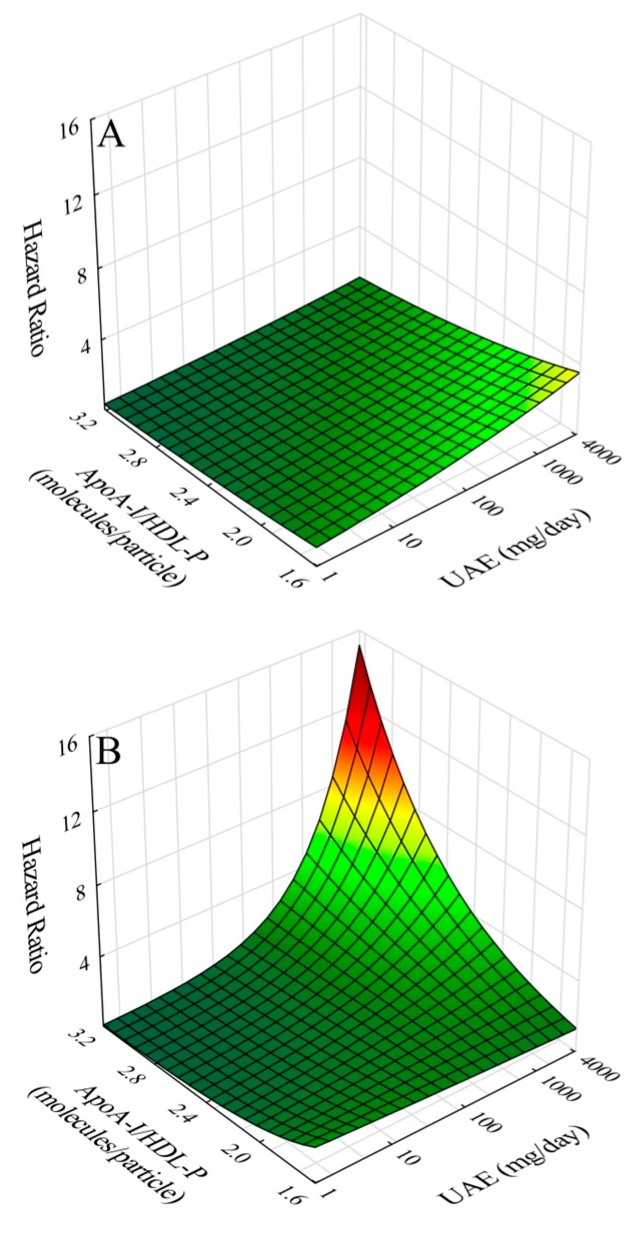
Hazard ratio for cardiovascular disease risk as a function of urinary albumin excretion (UAE) and mean HDL apoA-I content (ApoA-I/HDL-P) given as the number of apoA-I molecules per HDL particle over all HDL particles: (**A**) without inclusion of interaction of UAE and apoA-I content; and (**B**) with inclusion of interaction of UAE and apoA-I content.

**Figure 3:**
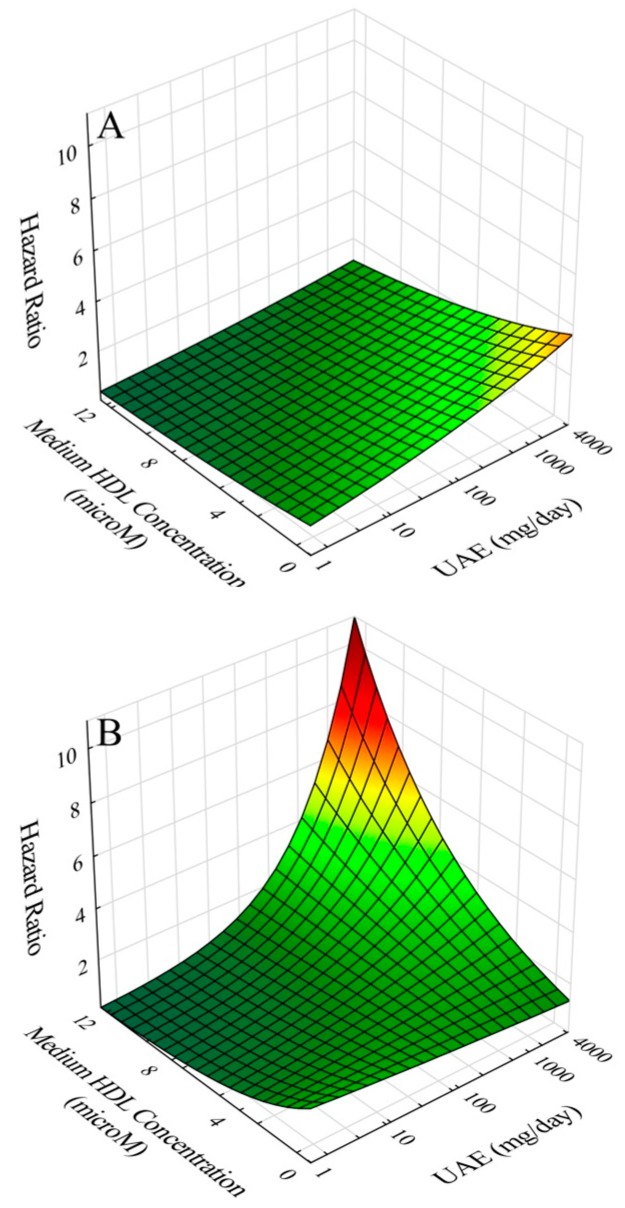
Hazard ratio for cardiovascular disease risk as a function of urinary albumin excretion (UAE) and medium-size HDL particle concentration: (**A**) without inclusion of interaction of UAE and HDL particle concentration; and (**B**) with inclusion of interaction of UAE and HDL particle concentration.

The effects of the addition of significant interaction terms are illustrated (see Appendix B for our approach to graphical modeling) for the ratio of apoA-I/HDL-P (Figure 2), and medium HDL-P (Figure 3), and for the nearly significant HDL-C interaction (Figure S1), and for the marginally significant HDL particle size interaction (Figure S2). In each case, the top panels show hazard ratio contours over relevant domains of UAE and HDL-associated parameters. In each case, increasing UAE shows increased risk, whereas increasing values of HDL-associated parameters are protective except for apoA-I/HDL-C. However, as illustrated in the bottom panels, when respective interaction terms in each of the cases are added to models, increasing UAE interacts with increasing HDL parameter values to intensify risk except for apoA-I/HDL-C where, instead, decreasing apoA-I/HDL-C interacts with increasing UAE to intensify risk.

### 2.5. Effects of Gender, Age, and Co-Morbidities on Outcomes and Interaction of UAE with ApoA-I/IHDL-P

Effects on outcome of the parameters included in the fully-adjusted model are given in Table 7. Hazard ratios reveal risk with increasing age, gender (males > females), apoB concentration, diabetes, past history of CVD, CRP concentration, and decreasing eGFR.

The effects of gender and age on the interaction of UAE with apoA-I/HDL-P were also examined. The results (HR, 95% CI, *p*-value) of analyses stratified by gender for the interaction term were significant for males (1.13, 1.02–1.26, *p* = 0.023) but non-significant for females. The results of analyses stratified by age (quartiles) for the interaction term were significant for quartile 4 (1.15, 1.00–1.32, *p* = 0.044) but non-significant for the three younger age quartiles. Thus, interaction of UAE with apoA-I/HDL-P was accentuated in males and in older individuals.

### 2.6. CVD Risk Relationships of ApoA-I/HDL-C and ApoA-I/IHDL-P

The apoA-I/HDL-C ratio was formulated as a crude estimate of HDL particle apoA-I content. The ratio was directly associated with CVD risk from results of both baseline (Table 2) and second screening (Table 5). Yet apoA-I/HDL-P, presumably also an estimator of HDL particle apoA-I content, instead revealed inverse correlation with risk. Appendix C, and Figures S3 and S4 present the rationale underlying this inverse relationship.

## 3. Discussion

The current work set-up as a dual-phase study utilizing multivariable risk modeling investigated incident CVD in a cohort of individuals of the PREVEND study as a function of UAE and measures of apoA-I content of HDL particles and concentrations of size-based HDL subfractions. In addition to confirming previous findings of risk associated with elevated UAE [1,2,3,4,5,6,7,8], baseline screening results using standard lipid and lipoprotein analyses revealed significant risk associations for HDL-C, apoA-I, and apoA-I/HDL-C. These findings were subsequently confirmed and extended using NMR lipoprotein analyses on samples collected four years later in risk models adjusted for gender, age, apoB, diabetes, past CVD history, CRP and eGFR. The results revealed an inverse association with risk for HDL-C, apoA-I, HDL-P, apoA-I/HDL-P, large HDL-P, and medium HDL-P, and a direct association for apoA-I/HDL-C. Moreover, in terms of interactions, further modeling revealed significant interactions between UAE and apoA-I/HDL-P and between UAE and medium HDL-P. In both cases, inclusion of interaction terms in risk models resulted in amplified risk at concurrently high levels of UAE and apoA-I/HDL-P and at concurrently high levels of UAE and concentration of medium-size HDL particles. We interpret these results to be consistent with our hypothesis that compositional features of HDL particles modulate the CVD risk associated with elevated UAE; furthermore, such findings may be indicative of pathogenic mechanisms shared by high UAE and compositional features of HDL particles in the development of CVD risk.

The results of our study demonstrated an independent protective effects against CVD risk for apoA-I/HDL-P. However, upon interaction with elevated UAE, the risk increased. This reversal is likely related to alterations in HDL structure and function in the setting of proteinuria resulting in pro-atherogenic transformation of HDL, especially as related to reverse cholesterol transport (RCT) [16]. Multiple factors associated with proteinuria have been shown to contribute to pro-atherogenic alterations in HDL that affect RCT by impairing maturation of HDL particles, particularly as related to cholesteryl ester enrichment. These include: 1. deficiency of lecithin-cholesteryl acyltransferase (LCAT) [17] as LCAT together with apoA-I mediates esterification of free cholesterol, although elevated LCAT activity has also been reported in proteinuria [18]; 2. hypoalbuminemia as albumin delivers significant amounts of free cholesterol from peripheral tissues to HDL particles, as it is a sign of inflammation [19]; 3. increased levels of cholesterol ester transfer protein (CETP) resulting in depletion of HDL particle cholesterol esters [20]; 4. decreased levels of the SRB1 hepatic HDL receptor resulting in decreased hepatic uptake of cholesteryl ester [21]; and 5. increased levels of the hepatic HDL endocytic receptor resulting in decreased apoA-I and HDL levels via increase in apoA-I mediated uptake of lipid-poor HDL [22]. In addition to proteinuria-associated RCT impairment, other atheroprotective HDL functions including anti-oxidant, anti-inflammatory, and anti-thrombotic activities would also likely be compromised with elevated UAE as in chronic renal disease [23,24,25]. Generation of dysfunctional HDL ensuing from these processes especially as affecting apoA-I would likely underlie the observed reversal of CVD risk association by apoA-I content of HDL from atheroprotective to atherogenic upon interaction with elevated UAE.

The results of our study also demonstrated independent protective effects against CVD risk by medium-size HDL particle concentration. However, again upon interaction with elevated UAE, the risk increased. Similar to apoA-I/HDL-P, medium HDL in the setting of elevated UAE could also develop into a source of pro-atherogenic HDL. Why this was not the case for concentrations of large and small HDL is not clear. Regarding large HDL, proportional hazards regression results with and without interaction were similar to those of medium HDL, except that the interaction term did not achieve statistical significance (*p* = 0.65) for large HDL. We speculate that this may relate to the generally greater estimated load of apoA-I carried by medium HDL particles (13.14 µM) in comparison to large HDL particles (8.12 µM) giving rise to a larger total pro-atherogenic load from medium HDL particles. The case for small-HDL particle concentration in UAE-associated risk was different in that interaction with elevated UAE did not achieve statistical significance.

In general consideration of HDL particle concentrations, the results of our study demonstrated that with increasing albuminuria, there were decreased concentrations of total, large, and medium size HDL particles, smaller HDL mean particle size, and increased concentration of small size HDL particles. These findings were generally consistent with the known lower levels of HDL_2_ and higher levels of HDL_3_ particles in nephrotic syndrome patients resulting from dysfunctional alterations of HDL secondary to impaired HDL particle maturation [16,26] and the lower total HDL particle concentration and lower mean HDL particle size in women with mild elevations in UAE [9].

Limitations in our study should be acknowledged. Although analysis results in the baseline screening round indicated potential for the apoA-II content of HDL to play a role in UAE-associated CVD risk, apoA-II levels in the second screening round were not available for confirmation. Additionally, data relating to other HDL constituents that likely play major roles in HDL metabolic, anti-inflammatory, and anti-thrombotic functionality were not available for assessment of interaction with UAE in CVD risk. In addition, the nature of the study did not allow us to delineate causal relationships in the complicated interplay between elevated UAE and aspects of HDL functionality that contribute to the process of atherogenesis leading to CVD. Moreover, actual experimental data relating directly to changes in HDL particle structure, and especially function, were not available. However, our approach of suing statistical interactions to reveal effects of HDL compositional characteristics in the context of increasing UAE on risk can be anticipated to be helpful in select subpopulations of subjects, in which documentation of HDL functionality and metabolomics studies may be particularly insightful. The strengths of our study included a design in which preliminary findings determined at study initiation from a large prospective population-based study (*N* = 6,286) could be extended from results of a second round of data collection on the same subjects approximately four years after study initiation. Furthermore, the second round of data collection afforded NMR-based lipid and lipoprotein analyses, including determinations of mean HDL particle size and concentrations of HDL-C, apoA-I, and HDL particle subfractions (large, medium, and small), with the use of a state-of-the-art deconvolution algorithm for the NMR data.

To summarize, the current study was undertaken to determine whether HDL particles share common pathogenic pathways with elevated UAE in development of CVD risk. The study involved proportional hazards multivariable modeling on subjects of PREVEND, a large prospective population-based study, assessing risk as a function UAE and of HDL-associated parameters that were determined by NMR lipid and lipoprotein analyses. The results demonstrated not only significant risk associations for elevated UAE and for multiple HDL-associated parameters, but moreover, and importantly, significant interactions of elevated UAE with apoA-I/HDL-P, a measure of HDL apoA-I content, and with concentration of medium size HDL particles. We believe that our study findings are consistent with HDL particles sharing pathogenic pathways with elevated UAE in CVD risk. Future studies should be oriented toward pathway characterizations.

## 4. Materials and Methods

### 4.1. Study Population

The study population was derived from PREVEND, a large general population-based, prospective cohort study begun in 1997 aiming to explore the role of albuminuria with CVD and renal disease [27]. Briefly, a questionnaire to all inhabitants of the city of Groningen, the Netherlands, aged 28–75 years (*N* = 85,421) was sent requesting an early morning urine specimen. The response rate was 48% (*N* = 40,856), of which 9,966 had urine albumin levels ≥10 mg/L, while 30,890 had urine albumin <10 mg/L. Pregnancy, type-1 type-2 diabetic individuals using insulin were exclusions. All subjects with urine albumin ≥10 mg/L (*N* = 7768) were combined with a random group of individuals with urine albumin <10 mg/L (*N* = 3395). Of this group, 8,592 individuals completed an additional screening program to comprise the PREVEND study cohort. Further exclusion of subjects without NMR lipoprotein analyses constituted the current study population (*N* = 6286). The PREVEND study was approved by the Medical Ethics Committee of the University of Groningen, the Netherlands, (Approval code 96/01/022, Approval date 23 February 1996). Informed written consent was obtained from all participants. The study adhered to the ethical principles set by the Declaration of Helsinki.

### 4.2. Clinical Parameters and Biomarkers

Cardiac history (hospitalization for myocardial infarction, revascularization procedures, or obstructive coronary artery disease) was acquired from questionnaires. Characterization of diabetes was either as fasting plasma glucose ≥7.0 mmol/L, self-report of physician diagnosis, or use of anti-diabetic medications. Medication use was retrieved via Groningen pharmacy dispensing data [28]. Smoking status was as either current smoker or not; ethanol use was either as one or less drinks per day or more than one drink per day. Body mass index (BMI) was calculated as weight divided by height squared (kg/m^2^). Blood pressure was measured in the supine position for 10 minutes at 1-minute intervals using automatic instrumentation (Dinamap XL Model 9300; Johnson-Johnson Medical, Tampa, FL, USA); reported values were means of the last two recordings [29].

UAE at study entry and second examination were determined as the mean of two 24-h collections over two consecutive days. Nephelometry (BNII, Dade Behring, Marburg, Germany) was used to measure urinary albumin concentration. Analyses of blood biomarker levels at study initiation were performed on overnight-fasted plasma and sera. Levels of creatinine, HDL-C, apoA-I, apoA-II, total cholesterol, triglycerides, apoB, glucose, and CRP were determined by standard techniques [30,31]. Estimated glomerular filtration rate (eGFR) was determined using the serum creatinine-based Chronic Kidney Disease Epidemiology Collaboration (CDK-EPI) equation [32]. NonHDL-C was calculated as the difference between total cholesterol and HDL-C. LDL-C levels were estimated using the Friedewald equation.

Concentrations of HDL-C, apoA-I, HDL-P, large HDL-P, medium HDL-P, and small HDL-P and HDL size were determined using nuclear magnetic resonance (NMR) spectroscopy on plasma samples collected at second screening. NMR MetaboProfile Analysis was used with the LP-4 deconvolution algorithm (LabCorp, Morrisville, NC, USA) for determination of analyte concentrations from NMR spectra collected on a Vantera Clinical Analyzer, as previously described [33,34]. Diameter range estimates for the NMR-derived HDL particle subfractions were as follows: large HDL (9.6–13 nm), medium HDL (8.1–9.5 nm), and small HDL (7.4–8.0 nm).

### 4.3. Outcome

Incident outcome events followed over time included CVD-related mortality and hospitalizations including acute MI, acute and subacute ischaemic heart disease, coronary artery bypass grafting, and percutaneous transluminal coronary angioplasty. Survival time was taken as date of initial urine collection (1997–1998) to date of first CV event or to 31 December 2008. For anyone lost to follow-up (396 of the overall cohort of 8592 participants), censoring date was date of removal from the municipal registry. Mortality data and causes of death were acquired by record linkage with the Dutch Central Bureau of Statistics. CVD morbidity data were obtained from PRISMANT, the Dutch national registry of hospital discharge diagnoses.

### 4.4. Data Analysis

Statistical and graphical analyses were performed using Statistica 10.0 (StatSoft, Inc., Tulsa, OK, USA). Results are reported as means ± SD for normally distributed variables and as medians (interquartile range) for non-normal variables. Differences among groups were assessed by chi-square testing for categorical variables and by ANOVA for continuous variables using Bonferroni correction for multiple comparisons. Non-normal variables were log-transformed for analyses. Multivariable Cox proportional hazards modeling was used to follow cardiovascular outcomes over time with continuous independent variables standardized by transformation to distributions with means of zero and standard deviations of one. Hazard ratios (HR) are per SD unit. Interactions between risk variables were assessed by the inclusion of multiplicative interaction terms into the base models. Two-sided *p*-values < 0.05 were considered statistically significant. The proportional hazards assumption was verified by correlation analysis of survival time with scaled Schoenfeld residuals.

## Figures and Tables

**Table 1:** Clinical covariates and biomarker levels (mean ± SD or median (interquartile range)) of the study population at the baseline evaluation stratified according to 24 h urinary albumin excretion (UAE) as: normoalbuminuria <30 mg/24 h, microalbuminuria between 30 and 300 mg/24 h, and macroalbuminuria >300 mg/24 h.

Parameter	Total Population	Normoalbuminuria (86.1% of Subjects)	Microalbuminuria	Macroalbuminuria	*p* ^a^	*p* ^b^	*p* ^c^
Subjects (%)	*N* = 6286	87.4	11.5	1.1			
Age (yrs)	48.7 ± 12.0	47.8 ± 11.8	54.6 ± 11.8	57.3 ± 12.4	<0.001		
Females (%)	51.0	53.2	36.8	29.6	<0.001		
Cardiac history (%)	3.0	2.1	7.2	10.3	<0.001		
Diabetes (%)	2.7	1.7	8.4	18.8	<0.001		
Statins (%)	3.7	3.0	7.3	13.2	<0.001		
Anti-hypertensives (%)	14.6	12.7	25.6	37.9	<0.001		
Current Smoker (%)	31.9	31.6	35.4	18.6	0		
Ethanol Use (%)	25.5	25.2	27.4	24.3	0.46		
BMI (kg/m^2^)	26.0 ± 4.1	25.7 ± 3.9	27.7 ± 4.6	29.5 ± 4.5	<0.001	<0.001	<0.001
Systolic BP (mmHg)	128 ± 19	126 ± 18	141 ± 23	151 ± 24	<0.001	<0.001	<0.001
Diastolic BP (mmHg)	74 ± 10	73 ± 9	79 ± 11	82 ± 10	<0.001	<0.001	<0.001
UAE (mg/24 h)	8.93 (6.19–15.61)	8.09 (5.92–11.93)	53.0 (38.0–87.1)	495 (347–1077)	<0.001	<0.001	<0.001
Creatinine (μmol/L)	83.4 ± 14.3	82.4 ± 13.3	88.2 ± 18.3	106.9 ± 33.9	<0.001	<0.001	<0.001
eGFR (mL/min/1.7 m^2^)	84.4 ± 15.0	85.2 ± 14.5	79.7 ± 16.5	67.7 ± 20.2	<0.001	<0.001	<0.001
HDL-C (mmol/L)	1.33 ± 0.40	1.35 ± 0.40	1.21 ± 0.38	1.15 ± 0.31	<0.001	<0.001	<0.001
ApoA-I (μmol/L)	48.0 ± 9.8	48.2 ± 9.8	46.9 ± 9.8	45.8 ± 9.9	<0.001	0.32	0.58
ApoA-II (μmol/L)	19.7 ± 3.7	19.7 ± 3.7	19.4 ± 3.9	18.1 ± 3.6	<0.001	0.013	0.027
ApoA-I/HDL-C (μmol/mmol)	37.6 ± 10.2	37.1 ± 7.9	40.7 ± 9.1	41.7 ± 10.2	<0.001	<0.001	<0.001
ApoA-II/HDL-C (μmol/mmol)	15.8 ± 4.6	15.6 ± 4.5	17.2 ± 5.1	16.7 ± 4.9	<0.001	<0.001	<0.001
Cholesterol (mmol/L)	5.62 ± 1.13	5.58 ± 1.12	5.89 ± 1.13	6.09 ± 1.35	<0.001	0.001	0.24
NonHDL-C (mmol/L)	4.28 ± 1.20	4.22 ± 1.19	4.67 ± 1.21	4.94 ± 1.39	<0.001	<0.001	0.005
LDL-C (mmol/L)	3.66 ± 1.04	3.62 ± 1.03	3.89 ± 1.04	3.98 ± 1.07	<0.001	0.18	0.40
Triglycerides (mmol/L)	1.14 (0.83–1.66)	1.11 (0.81–1.60)	1.43 (1.00–2.20)	1.49 (1.07–2.77)	<0.001	<0.001	<0.001
ApoB (g/L)	1.03 ± 0.30	1.01 ± 0.29	1.12 ± 0.32	1.15 ± 0.30	<0.001	<0.001	0.024
Glucose (mmol/L)	4.83 ± 1.08	4.75 ± 0.87	5.37 ± 1.81	5.80 ± 2.70	<0.001	<0.001	<0.001
CRP (mg/L)	1.18 (0.53–2.77)	1.07 (0.49–2.55)	2.08 (0.96–4.32)	2.57 (1.25–6.24)	<0.001	<0.001	<0.001

^a^ Significance levels from chi square testing for categorical variables and unadjusted ANOVA for continuous variables. Post-hoc testing revealed significant differences between normoalbuminuric and microalbuminuric subjects for all parameters except apoA-II; significant differences between normoalbuminuric and macroalbuminuric subjects for all parameters except apoA-I and apoA-II/HDL-C; and significant differences between microalbuminuric and macroalbuminuric subjects for BMI, systolic blood pressure, creatinine, eGFR, apoA-II, triglycerides, and glucose. ^b^ Significance levels from ANOVA adjusted for gender and age. ^c^ Significance levels from ANOVA adjusted for gender, age, apoB, diabetes, past CVD history, and eGF.

**Table 2:** Interaction of urinary albumin excretion (UAE) with HDL-related parameters for baseline evaluation data as assessed by Cox proportional hazards models of cardiovascular event occurrence adjusted for gender and age. Hazard ratios are per SD unit for all variables.

Models	Model without Interaction			Model with Interaction		
Parameters	HR	95% CI	*p*	HR	95% CI	*p*
UAE	1.11	1.00–1.23	0.036	1.16	1.02–1.31	0.023
HDL-C	0.62	0.53–0.74	<10^−6^	0.61	0.51–0.73	<10^−6^
interaction				1.07	0.94–1.22	0.34
UAE	1.15	1.04–1.27	0.005	1.16	1.04–1.29	0.006
ApoA-I	0.77	0.67–0.88	<10^−3^	0.76	0.66–0.88	<10^−3^
interaction				1.02	0.93–1.11	0.72
UAE	1.16	1.05–1.28	0.003	1.16	1.05–1.29	0.004
ApoA-II	0.89	0.78–1.01	0.067	0.88	0.77–1.01	0.068
interaction				1.01	0.93–1.11	0.76
UAE	1.11	1.01–1.23	0.036	1.17	1.05–1.30	0.003
ApoA-I/HDL-C	1.26	1.14–1.39	<10^−5^	1.32	1.19–1.47	<10^−6^
interaction				0.91	0.84–0.99	0.019
UAE	1.11	1.01–1.23	0.035	1.15	1.04–1.28	0.009
ApoA-II/HDL-C	1.34	1.20–1.51	<10^−6^	1.40	1.24–1.58	<10^−6^
interaction				0.93	0.85–1.01	0.10

**Table 3:** Interaction of UAE with HDL-related parameters for baseline evaluation data as assessed by Cox proportional hazards models of cardiovascular event occurrence concurrently adjusted for gender, age, apoB concentration, diabetes, past CVD history, C-reactive protein (CRP) concentration, and eGFR.

Models	Model without Interaction			Model with Interaction		
Parameters	HR	95% CI	*p*	HR	95% CI	*p*
UAE	1.04	0.94–1.16	0.46	1.10	0.97–1.26	0.14
HDL-C	0.74	0.61–0.88	<10^−3^	0.72	0.59–0.86	<10^−3^
interaction				1.10	0.96–1.26	0.16
UAE	1.07	0.96–1.19	0.21	1.07	0.95–1.19	0.26
ApoA-I	0.73	0.62–0.85	<10^−4^	0.73	0.662–0.86	<10^−3^
interaction				1.00	0.89–1.12	0.96
UAE	1.06	0.96–1.18	0.26	1.07	0.96–1.19	0.22
ApoA-II	0.76	0.65–0.89	<10^−3^	0.75	0.64–0.89	<10^−3^
interaction				1.03	0.92–1.14	0.63
UAE	1.04	0.94–1.16	0.44	1.11	0.99–1.25	0.078
ApoA-I/HDL-C	1.10	0.96–1.26	0.16	1.16	1.01–1.34	0.036
interaction				0.90	0.81–0.99	0.025
UAE	1.05	0.94–1.16	0.41	1.07	0.96–1.20	0.23
ApoA-II/HDL-C	1.16	1.00–1.34	0.52	1.19	1.02–1.39	0.029
interaction				0.95	0.86–1.05	0.29

**Table 4:** NMR-derived biomarker levels (mean ± SD) in the study population at second screening round stratified according to 24 h urinary albumin excretion (UAE) as: normoalbuminuria <30 mg/24 h, microalbuminuria between 30 and 300 mg/24 h, and macroalbuminuria >300 mg/24 h. Ranges of HDL particle diameters were: large HDL (9.6–13 nm), medium HDL (8.1–9.5 nm), and small HDL (7.4–8.0 nm).

Parameter	Total Population	Normoalbuminuria (86.1% of subjects)	Microalbuminuria	Macroalbuminuria	*p* ^a^	*p* ^b^	*p* ^c^
HDL-P (μmol/L)	21.14 ± 2.74	21.23 ± 2.71	20.57 ± 2.83	20.40 ± 3.23	<0.001	<0.001	0.003
Large HDL-P (μmol/L)	1.81 ± 1.29	1.86 ± 1.30	1.50 ± 1.21	1.49 ± 1.16	<0.001	0.001	0.009
Medium HDL-P (μmol/L)	5.15 ± 2.19	5.25 ± 2.19	4.59 ± 2.11	4.06 ± 2.09	<0.001	<0.001	0.18
Small HDL-P (μmol/L)	14.18 ± 2.87	14.13 ± 2.88	14.48 ± 2.75	14.85 ± 3.06	<0.001	0.65	0.53
HDL Size (nm)	8.97 ± 0.45	8.99 ± 0.45	8.84 ± 0.45	8.82 ± 0.45	<0.001	<0.001	0.005
HDL-C (mmol/L)	1.33 ± 0.32	1.34 ± 0.31	1.23 ± 0.32	1.21 ± 0.34	<0.001	<0.001	0.009
ApoA-I (μmol/L)	45.1 ± 7.8	45.4 ± 7.7	43.0 ± 7.9	42.5 ± 8.6	<0.001	<0.001	0.023
ApoA-I/HDL-C (μmol/mmol)	34.5 ± 2.9	34.4 ± 2.9	35.5 ± 3.0	35.9 ± 3.1	<0.001	<0.001	<0.001
ApoA-I/HDL-P (molecules/particle)	2.13 ± 0.21	2.14 ± 0.21	2.09 ± 0.20	2.08 ± 0.21	<0.001	0.25	0.38

^a^ Significance levels from unadjusted ANOVA. Post-hoc testing revealed significant differences between normoalbuminuric and microalbuminuric subjects for all parameters; significant differences between normoalbuminuric and macroalbuminuric subjects for all parameters except small HDL-P and ApoA-I/HDL-P; and no significant differences between microalbuminuric and macroalbuminuric subjects for any of the parameters. ^b^ Significance levels from ANOVA adjusted for gender and age. ^c^ Significance levels from ANOVA adjusted for gender, age, apoB, diabetes, past CVD history, and eGFR. Abbreviations: HDL-P, total HDL particle concentration; Large HDL-P, large HDL particle concentration; Medium HDL-P, medium HDL particle concentration; and Small HDL-P, small HDL particle concentration.

**Table 5:** Interaction of UAE with HDL-related parameters for second screening round as assessed by Cox proportional hazards models of cardiovascular event occurrence adjusted for gender and age. Hazard ratios are per SD unit for all variables.

Models	Model without Interaction			Model with Interaction		
Parameters	HR	95% CI	*p*	HR	95% CI	*p*
UAE	1.21	1.12–1.32	<10^−5^	1.23	1.12–1.34	<10^−5^
HDL-P	0.83	0.75–0.93	0.001	0.82	0.73–0.93	0.004
interaction				1.02	0.95–1.10	0.56
UAE	1.21	1.11–1.31	<10^−5^	1.23	1.13–1.34	<10^−5^
Large HDL-P	0.74	0.64–0.85	<10^−4^	0.72	0.62–0.84	<10^−4^
interaction				1.05	0.96–1.16	0.30
UAE	1.20	1.10–1.30	<10^−4^	1.28	1.16–1.40	<10^−5^
Medium HDL-P	0.74	0.65–0.84	<10^−5^	0.70	0.61–0.81	<10^−5^
interaction				1.11	1.01–1.22	0.029
UAE	1.22	1.13–1.33	<10^−5^	1.24	1.14–1.35	<10^−6^
Small HDL-P	1.11	0.99–1.24	0.07	1.15	1.02–1.30	0.024
interaction				0.93	0.86–1.01	0.10
UAE	1.20	1.10–1.30	<10^−4^	1.24	1.13–1.36	<10^−5^
HDL Size	0.70	0.61–0.80	<10^−6^	0.67	0.58–0.77	<10^−6^
interaction				1.08	0.99–1.18	0.10
UAE	1.19	1.10–1.29	<10^−4^	1.29	1.14–1.37	<10^−5^
HDL-C	0.69	0.60–0.79	<10^−6^	0.66	0.57–0.76	0.004
interaction				1.08	1.00–1.18	0.06
UAE	1.20	1.11–1.30	<10^−4^	1.24	1.14–1.37	<10^−5^
ApoA-I	0.74	0.65–0.84	<10^−5^	0.71	0.62–0.81	<10^−5^
interaction				1.07	0.99–1.16	0.11
UAE	1.19	1.09–1.29	<10^−4^	1.23	1.12–1.35	<10^−5^
ApoA-I/HDL-C	1.37	1.22–1.55	<10^−6^	1.43	1.25–1.62	<10^−6^
interaction				0.94	0.86–1.02	0.11
UAE	1.21	1.11–1.31	<10^−5^	1.26	1.15–1.37	<10^−6^
ApoA-I/HDL-P	0.74	0.65–0.85	<10^−4^	0.71	0.61–0.82	<10^−5^
interaction				1.10	1.00–1.20	0.039

HDL-P, total HDL particle concentration; Large HDL-P, large HDL particle concentration; Medium HDL-P, medium HDL particle concentration; and Small HDL-P, small HDL particle concentration.

**Table 6:** Interaction of UAE with HDL-related parameters for second screening round as assessed by Cox proportional hazards models of cardiovascular event occurrence adjusted for gender, age, apoB, diabetes, past CVD history, CRP, and eGFR. Hazard ratios are per SD unit for all variables.

Models	Model without Interaction			Model with Interaction		
Parameters	HR	95% CI	*p*	HR	95% CI	*p*
UAE	1.13	1.05–1.24	0.003	1.16	1.076–1.27	0.002
HDL-P	0.88	0.79–0.99	0.030	0.86	0.77–0.968	0.018
interaction				1.04	0.96–1.12	0.36
UAE	1.14	1.05–1.24	0.003	1.16	1.06–1.27	0.001
Large HDL-P	0.86	0.74–1.01	0.060	0.84	0.72–0.99	0.037
interaction				1.05	0.96–1.16	0.28
UAE	1.13	1.04–1.243	0.006	1.21	1.09–1.33	<10^−3^
Medium HDL-P	0.801	0.71–0.92	0.002	0.76	0.66–0.88	<10^−3^
interaction				1.12	1.02–1.23	0.018
UAE	1.14	1.05–1.24	0.003	1.15	1.06–1.26	0.001
Small HDL-P	1.05	0.93–1.18	0.44	1.08	0.95–1.23	0.24
interaction				0.94	0.87–1.03	0.19
UAE	1.13	1.04–1.24	0.004	1.17	1.07–1.29	<10^−3^
HDL Size	0.80	0.69–0.92	0.002	0.77	0.66–0.90	<10^−3^
interaction				1.07	0.98–1.17	0.14
UAE	1.13	1.04–1.24	0.004	1.18	1.08–1.31	<10^−3^
HDL-C	0.79	0.68–0.91	0.002	0.76	0.65–0.88	<10^−3^
interaction				1.08	1.00–1.187	0.049
UAE	1.14	1.04–1.24	0.003	1.18	1.08–1.30	<10^−3^
ApoA-I	0.83	0.73–0.95	0.006	0.80	0.70–0.92	0.002
interaction				1.08	0.99–1.17	0.08
UAE	1.13	1.04–1.23	0.006	1.17	1.06–1.29	0.002
ApoA-I/HDL-C	1.23	1.08–1.40	0.002	1.27	1.10–1.45	<10^−3^
interaction				0.94	0.87–1.03	0.18
UAE	1.14	1.05–1.24	0.003	1.18	1.08–1.30	<10^−3^
ApoA-I/HDL-P	0.86	0.74–1.00	0.044	0.81	0.69–0.95	0.011
interaction				1.09	1.00–1.20	0.048

HDL-P, total HDL particle concentration; Large HDL-P, large HDL particle concentration; Medium HDL-P, medium HDL particle concentration; and Small HDL-P, small HDL particle concentration.

**Table 7:** Risk model results of second screening round examining interaction of UAE with apoA-I/HDL-C with adjustments for gender, age, apoB, diabetes, past CVD history, CRP, and eGFR. Hazard ratios are per SD unit for continuous variables.

Parameters	HR	95% CI	*p*
Gender	2.16	1.65–2.83	<10^−6^
Age	1.85	1.59–2.14	<10^−6^
ApoB Concentration	1.22	1.08–1.37	<10^−3^
Diabetes	1.61	1.08–2.40	0.020
Past CVD History	2.27	1.64–3.15	<10^−5^
CRP Concentration	1.18	1.03–1.34	0.013
eGFR	0.88	0.78–1.00	0.053
UAE	1.18	1.08–1.30	<10^−3^
ApoA-I/HDL-C	0.81	0.69–0.95	0.011
UAE x ApoA-I/HDL-P	1.09	1.00–1.20	0.048

ApoB, apolipoprotein B; CRP, C-reactive protein; eGFR, estimated glomerular filtration rate; UAE, urinary albumin excretion; and apoA-I, apolipoprotein A-I.

## Supplementary Materials

Supplementary materials can be found at https://www.mdpi.com/1422-0067/20/4/977/s1. **Figure S1.** Hazard ratio for cardiovascular disease risk as a function of urinary albumin excretion (UAE) and HDL-C: (A) without inclusion of interaction of UAE and HDL particle concentration; and (B) with inclusion of interaction of UAE and HDL particle concentration. **Figure S2.** Hazard ratio for cardiovascular disease risk as a function of urinary albumin excretion (UAE) and mean HDL particle size (nm): A. without inclusion of interaction of UAE and HDL particle size; and B. with inclusion of interaction of UAE and HDL particle size. **Figure S3.** Scatter plot of apoA-I/HDL-P versus apoA-I/HDL-C. **Figure S4.** Hazard ratio for cardiovascular disease risk as a function of UAE and apoA-I/HDL-C: A. without inclusion of interaction of UAE and apoA-I/HDL-C; and B. with inclusion of interaction of UAE and apoA-I/HDL-C.

## Appendix A. Estimates of Mean Number of apoA-I Molecules per Particle and Mean apoA-I Concentrations of Large, Medium, and Small HDL Subfractions

Lastly, multiple regression analysis with total apoA-I concentration as a function of large, medium, and small particles was used to estimate the mean number of apoA-I molecules/particle for the three subfractions. From these values and the total particle concentrations of the subfractions, the concentrations of apoA-I in each of the three subfractions were estimated. Parameters are defined as follows:ApoA1 = total apoA-I concentration (μM), L = large HDL particle concentration (μM), M = medium HDL particle concentration (μM), S = small HDL particle concentration (μM), *N*_L_ = mean number of apoA-I molecules per large HDL particle, *N*_M_ = mean number of apoA-I molecules per medium HDL particle, and *N*_S_ = mean number of apoA-I molecules per small HDL particle.

The mass balance equation for total apoA-I concentration is as follows:

ApoA1 = *N*_L_ × L + *N*_M_ × M + *N*_S_ × S

ApoA1, L, M, and S were measured for each patient in the study. Thus, multiple regression analysis can be performed with apoA1 as a function of L, M, and S. The resulting regression coefficients will represent estimates of *N*_L_, *N*_M_, and *N*_S_ which can then be multiplied by L, M, and S, respectively to generate the apoA-I concentrations of each of the subfractions.

Results for number of apoA-I molecules per particle were as follow: large HDL: 4.49 ± 0.014, *p* < 10^−6^; medium: 2.55 ± 0.007, *p* < 10^−6^; and small HDL: 1.68 ± 0.003, *p* < 10^−6^). Results for apoA-I concentration were: large HDL: 8.12 ± 0.073 μM, medium HDL: 13.14 ± 0.070 μM, and small HDL: 23.83 ± 0.061 μM.

## Appendix B. Approach to Graphical Modeling of CVD Risk Contours without and with Interactions

Illustrations of interactions of UAE with HDL-associated continuous variables were performed analytically from Cox regression models to give contour plots of HR as a function of UAE and HDL-associated parameters. Using apoA-I as an example, this would involve evaluation of the expression for HR as:

exp(β_UAE_ × UAE + β_apoA-I_ × apoA-I + β_interact_ × UAE × apoA-I)

Results are presented as 3-dimensional plots showing HR as a surface extending over clinically relevant domains of UAE and HDL-associated parameters.

## Appendix C. Rationale for inverse correlation of apoA-I/HDL-P with apoA-I/HDL-C

To investigate the inverse correlation, a scatter plot of apoA-I/HDL-P versus apoA-I/HDL-C was generated (Figure S3). The plot revealed an inverse relationship between the two (*r* = −0.78, *p* < 0.0001) consistent with the CVD risk association results.

To explore effects of interaction of apoA-I/HDL-C with UAE, plots of risk as a function of UAE and apoA-I/HDL-C without and with interaction between the two were generated (Figure S4) noting that the interaction term between UAE and apoA-I/HDL-C demonstrated only a marginal approach to significance (Table 4; HR = 0.94, *p* = 0.11). The risk plot without interaction demonstrated additive effects on risk for increasing UAE and increasing apoA-I/HDL-C. The risk plot with interaction demonstrated mainly attenuation of the direct association of apoA-I/HDL-C with risk.

Regarding the basis for the inverse correlation, our underlying assumption borne out by correlation analysis regarding apoA-I concentration is that there is a direct linear relationship of apoA-I concentration with HDL-P (*r* = 0.83, *p* < 10^−6^) and that there is a direct linear relationship of apoA-I with HDL-C (*r* = 0.95, *p* < 10^-6^). Thus,

ApoA-I = *k*_1_ × HDL-P and ApoA-I = *k*_2_ × HDL-C

Rearranging the equations gives,

ApoA-I/HDL-P = *k*_1_ and ApoA-I/HDL-C = *k*_2_

Adding together the two previous equations gives,

ApoA-I/HDL-P + ApoA-I/HDL-C = *k*_1_ + *k*_2_

Solving the previous equation for ApoA-I/HDL-P gives,

ApoA-I/HDL-P = (*k*_1_ + *k*_2_) − ApoA-I/HDL-C

We conclude that ApoA-I/HDL-P and ApoA-I/HDL-C have an inverse relationship.
