# Supplementary material for: Compositional Features of HDL Particles Interact with Albuminuria to Modulate Cardiovascular Disease Risk
Source: Int J Mol Sci. 2019 Feb 23;20(4):977. doi: 10.3390/ijms20040977 (PMC6412699; doi:10.3390/ijms20040977)
Supplement: Supplementary file 1 [file ijms-20-00977-s001.pdf]

International Journal of
Molecular Sciences

MDPI

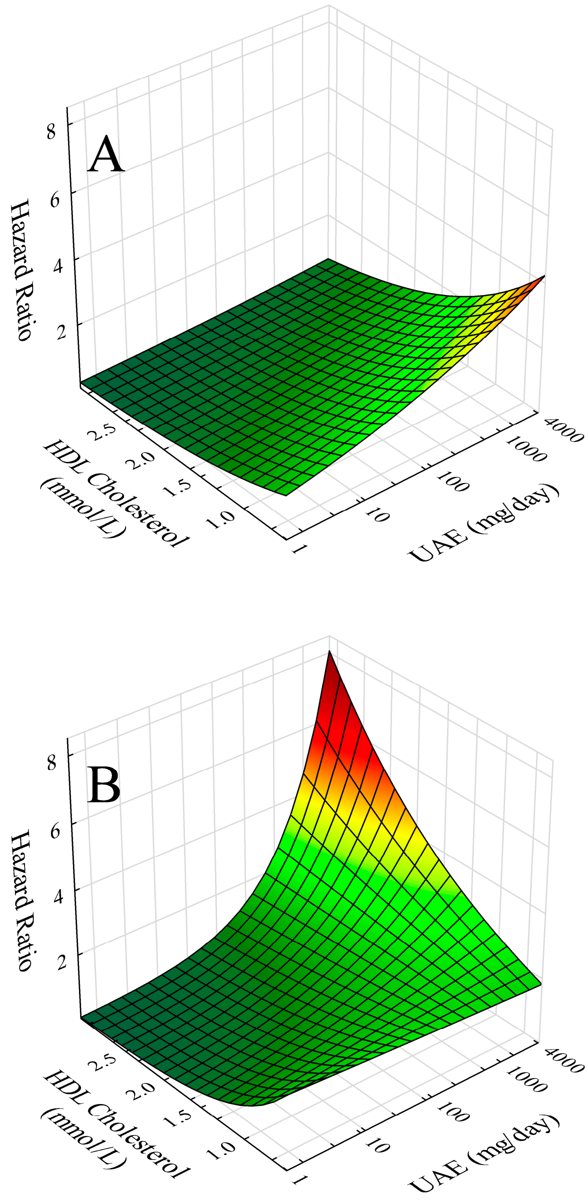

Figure S1. Hazard ratio for cardiovascular disease risk as a function of urinary albumin excretion (UAE) and HDL-C: (**A**) without inclusion of interaction of UAE and HDL particle concentration; and (**B**) with inclusion of interaction of UAE and HDL particle concentration.

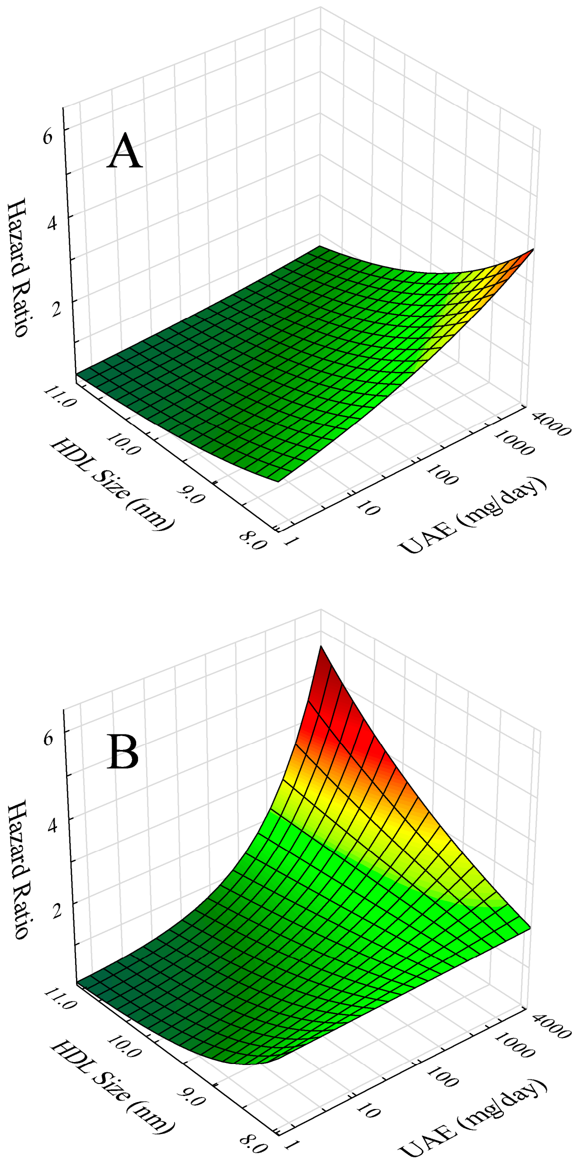

Figure S2. Hazard ratio for cardiovascular disease risk as a function of urinary albumin excretion (UAE) and mean HDL particle size (nm): A. without inclusion of interaction of UAE and HDL particle size; and B. with inclusion of interaction of UAE and HDL particle size.

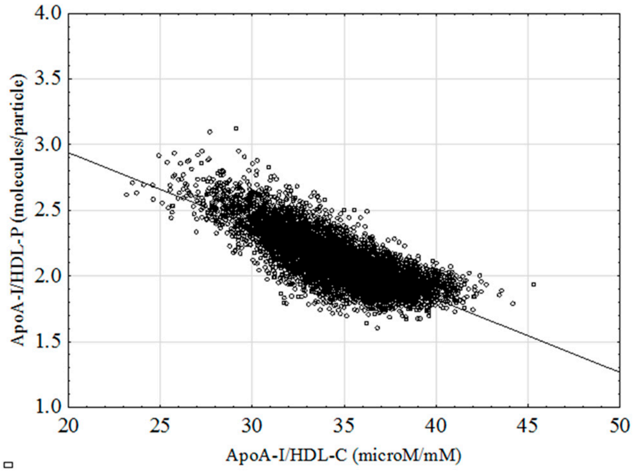

Figure S3. Scatter plot of apoA-I/HDL-P versus apoA-I/HDL-C. Regression: ApoA1/HDL-P = 4.0546 –0.0558 × ApoA-I/HDL-C, $r = -0.78$, $p < 0.0001$.

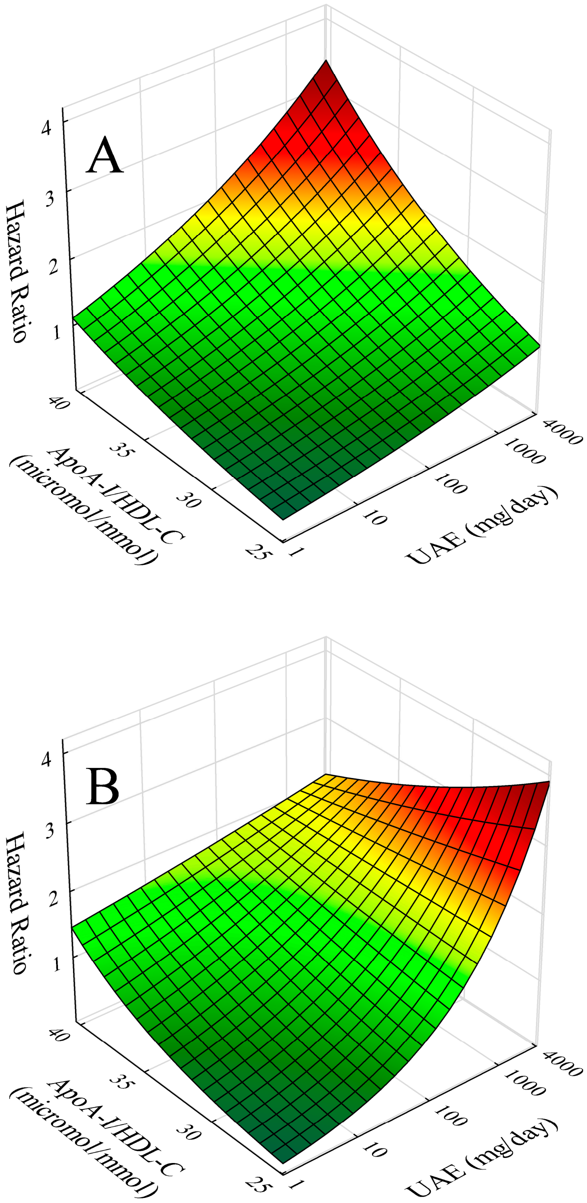

Figure S4. Hazard ratio for cardiovascular disease risk as a function of UAE and apoA-I/HDL-C: A. without inclusion of interaction of UAE and apoA-I/HDL-C; and B. with inclusion of interaction of UAE and apoA-I/HDL-C.
